# Transient Cardiac Dysfunction Following a Cerebrovascular Accident

**DOI:** 10.7759/cureus.16023

**Published:** 2021-06-29

**Authors:** Steven Hamilton, Rana A Tauseen, Sara L Wallach, Adam C Kaplan

**Affiliations:** 1 Internal Medicine, Jersey Shore University Medical Center/Saint Francis Medical Center Program, Trenton, USA

**Keywords:** cerebral t waves, cerebro-vascular accident (stroke), t-waves, left ventricular systolic dysfunction, cardiac arrythmia

## Abstract

Acute cerebral injuries are often accompanied by sudden electrocardiogram (ECG) changes such as cardiac arrhythmias, QT prolongation, and abnormal T-wave morphology. One rare phenomenon is “cerebral T-waves”, which are T-waves observed in the context of stroke and described as transient, symmetric, and deeply inverted. The classic cerebral T wave is defined as a T-wave inversion of ≥5 mm depth in at least four contiguous precordial leads, and it is more commonly observed in the setting of acute ischemic stroke rather than hemorrhagic stroke. We describe the case of a patient who initially presented with acute pulmonary edema, T-wave inversions in the precordial leads, and left ventricular dysfunction on echocardiogram raising suspicion of an ischemic cardiac event. However, a brain CT scan performed on the third day of admission proved us wrong.

## Introduction

T-wave changes on ECG may be the result of various conditions that might be cardiac or non-cardiac in origin. The differential diagnosis for these changes may include cardiovascular as well as neurologic involvement. Cardiovascular etiologies of T-wave changes include myocardial ischemia, myocardial infarction, myocarditis, pericarditis, and ventricular hypertrophy with strain pattern. Neurologic causes include subarachnoid hemorrhage, subdural hematoma, and acute cerebrovascular accidents (CVAs) [1]. Cerebral ischemic or hemorrhagic events have been associated with various ECG changes. These changes are usually transient. Cerebral T waves, which are defined as deep symmetrical inverted T waves seen in the pericardial leads, are the most frequently noticed changes. The exact mechanism for such changes is not well established yet but a previous study hypothesized that possible injury to the insular cortex results in an increase in sympathetic tone to the cardiac system [2]. One recent retrospective study even went beyond the transient ECG changes and linked acute cerebral injuries to transient cardiac dysfunction, such as reduced systolic function, due to autonomic dysfunction, expanding on the effects of the hypothesis above [3].

## Case presentation

**Figure 1:**
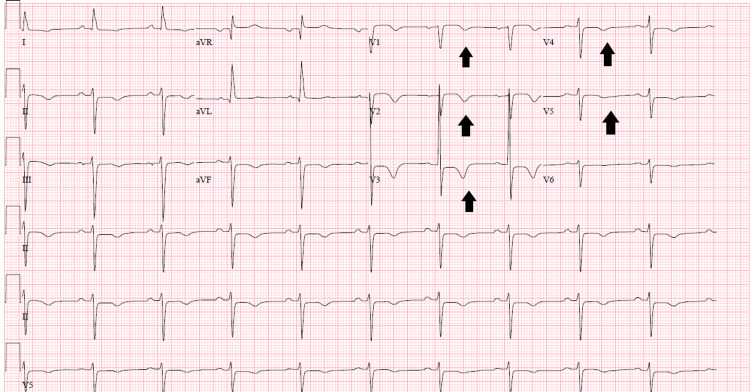
ECG showing T-wave inversions over the pericordial leads (black arrows).

We present the case of a 75-year-old male patient with a past medical history of diabetes mellitus and unremarkable family history who presented to our emergency department (ED) after sudden onset shortness of breath and sweating witnessed by his friend. During transport to the hospital, the patient’s shortness of breath worsened with accompanying hypoxia. Conservative measures to support his respiration failed and he was intubated in the field. In ED, the patient was found to be hypertensive with a blood pressure of 173/92 mmHg, heart rate of 82 beats/min, diffuse inspiratory fine crackles on auscultation with markedly elevated venous jugular distention. The rest of the physical examination was unremarkable, though limited in its scope since the patient was sedated and intubated. Discussion with his friend did not reveal a history significant for chest pain at the time of the symptom onset. Blood work was remarkable for highly elevated B-type natriuretic peptide (BNP) at 2943 pg/mL (< 100 pg/mL) and troponin I at 0.09 ng/mL (<0.03 ng/mL), hemoglobin A1c at 7.1 %, total cholesterol at 153 mg/dL, low-density lipoprotein at 111 mg/dL; the urine drug screen was negative. The remainder of his blood work was unremarkable. In the ED, the chest x-ray was remarkable for increased bronchovascular markings suggestive of pulmonary edema, ECG showed T-wave inversions in the pericordial leads from V1 to V5 (Figure 1), CT scan of the brain without contrast was unremarkable for any ischemic or hemorrhagic pathology.

Due to his initial presentation with acute pulmonary edema, elevated troponin levels, and T-wave inversions in the ECG, the patient was treated for acute coronary syndrome with aspirin, high-intensity statin, intravenous heparin drip, and intravenous diuretics. Troponin follow-up showed an uptrend with a peak at 1.88 ng/mL. Emergent echocardiography showed moderately reduced left ventricular systolic function, ejection fraction of 40-45%, with hypokinesis-akinesis of the mid and basal inferior wall segment with no evidence of left ventricular wall thrombus. Due to the critical nature of the patient cardiology deferred coronary angiography until the patient was more stable. However, on the third day of the hospital course, a detailed neurological exam after extubating revealed a focal neurological deficit in the form of motor power 2 out of 5 affecting proximal and distal muscle groups of his right upper and lower extremities. Areflexia and positive Babinski sign were also noted.

**Figure 2:**
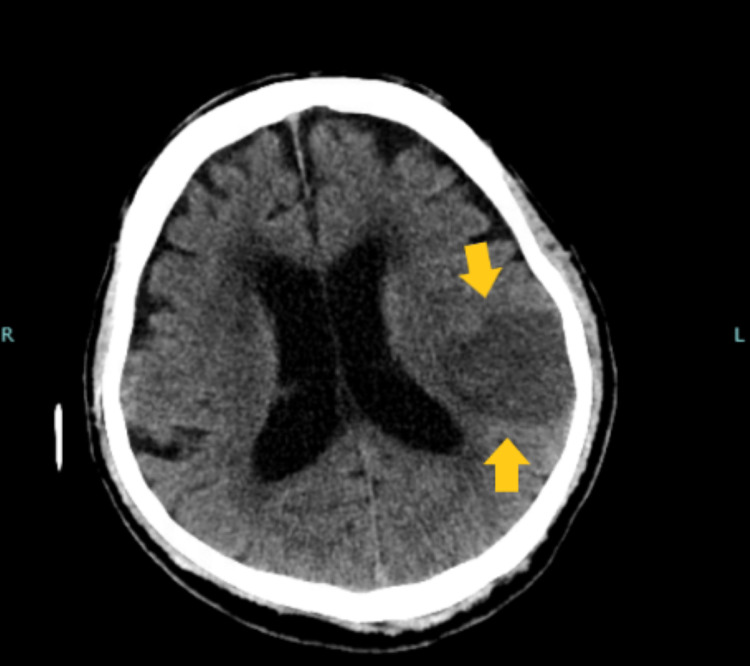
CT scan on the second day of admission showing large evolving infarct within left temporal and left parietal lobe (yellow arrows).

**Figure 3:**
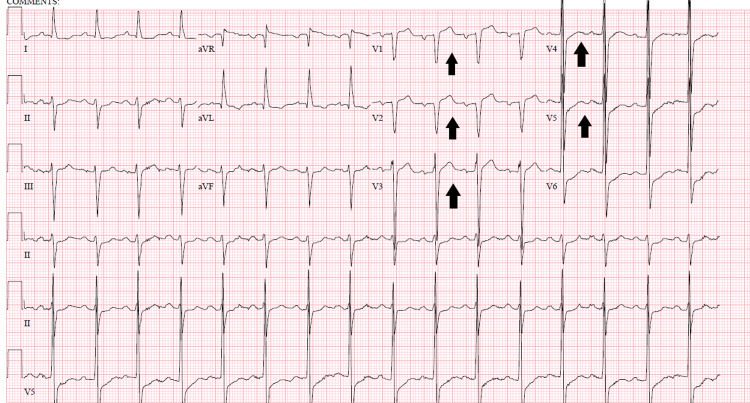
ECG showing the resolution of the T-wave inversions on the third day of hospital stay (black arrows).

Secondary to this finding a CT scan of the head was ordered which revealed a large evolving infarct within left the temporal/parietal lobe (Figure 2). Interestingly, repeat ECGs showed resolution of the previously identified T-wave inversions (Figure 3). Upon multidisciplinary discussion, it was determined his clinical presentation, particularly the reversible T waves in four contiguous leads and wall motion abnormalities were likely due to his large ischemic infarct and not likely due to the acute coronary syndrome. Therefore the patient did not undergo coronary angiography during his hospitalization. Unfortunately, the patient was lost to follow-up.

## Discussion

Cerebral T waves and left ventricular (LV) dysfunction are rare findings in patients with acute cerebrovascular events. A retrospective study done by Stone et al. showed that in 800 patients with an acute cerebrovascular accident, only 17 patients were found to have cerebral T waves on ECG with the majority of cases involving the middle cerebral artery (MCA) (11 patients out of the 17). Furthermore, they went on to describe three patients with concurrent LV wall motion abnormalities and cerebral T waves, who all had infarcts in the MCA distribution, similar to our patient’s findings [3].

While there is no argument that a patient presenting with pulmonary edema, ECG changes, and elevated troponins should be evaluated for acute coronary syndrome, we believe it is important to recognize other etiologies so that appropriate treatment can be started and treatment harm avoided. Examples of such harms may be the rapid lowering of blood pressure or the initiation of anticoagulation, which are indicated in acute coronary syndrome but not indicated for an acute ischemic stroke. The lack of chest pain, only a moderate rise in troponin, and resolution of the T wave changes indicated the possibility of other etiologies for our patient that only became clear upon follow-up exams revealing a focal neurologic deficit, which was confirmed with a CT scan of the head.

The proposed pathophysiology behind an acute cerebrovascular event and cardiac dysfunction was described by Rogers et al. In their study, they stimulated the left stellate ganglion which in turn produce deeply inverted T waves [4]. This finding paired with the knowledge that the insular cortex plays a role in autonomic regulation, which when disrupted as in the case of an MCA infarct, can lead to the cardiac abnormalities seen in our patient.

## Conclusions

Cerebral T waves and LV dysfunction are rare presenting signs in cerebrovascular injuries. However, their recognition is extremely important to mitigate unnecessary treatment that may be harmful to the patient. Recognizing such signs may help in identifying the true etiology, such as a stroke and help in the initiation of stroke care before evidence from CT scans, which can be delayed.
